# Therapeutic Consequences and Prognostic Impact of Multimorbidity in Heart Failure: Time to Act

**DOI:** 10.3390/jcm14010139

**Published:** 2024-12-29

**Authors:** Fanni Bánfi-Bacsárdi, Ádám Kazay, Tamás G. Gergely, Zsolt Forrai, Tamás Péter Füzesi, Laura Fanni Hanuska, Pál Péter Schäffer, Dávid Pilecky, Máté Vámos, Vivien Vértes, Miklós Dékány, Péter Andréka, Zsolt Piróth, Noémi Nyolczas, Balázs Muk

**Affiliations:** 1 Department of Adult Cardiology, Gottsegen National Cardiovascular Center, 1096 Budapest, Hungary; 2 Doctoral School of Clinical Medicine, University of Szeged, 6720 Szeged, Hungary; 3 Department of Cardiology, Central Hospital of Northern Pest—Military Hospital, 1134 Budapest, Hungary; 4 Cardiac Electrophysiology Division, Cardiology Center, Internal Medicine Clinic, University of Szeged, 6725 Szeged, Hungary; 5 Károly Rácz Doctoral School of Clinical Medicine, Semmelweis University, 1085 Budapest, Hungary

**Keywords:** heart failure, multimorbidity, comorbidity, prognosis, guideline-directed medical therapy

## Abstract

**Background/Objectives**: In heart failure (HF) with reduced ejection fraction (HFrEF), the early diagnosis and proper treatment of comorbidities (CMs) are of fundamental relevance. Our aim was to assess the prevalence of CMs among real-world patients requiring hospitalisation for HFrEF and to investigate the effect of CMs on the implementation of guideline-directed medical therapy (GDMT) and on all-cause mortality (ACM). **Methods**: The data of a consecutive HFrEF patient cohort hospitalised for HF between 2021 and 2024 were analysed retrospectively. Sixteen CMs (6 CV and 10 non-CV) were considered. Patients were divided into three categories: 0–3 vs. 4–6 vs. ≥7 CMs. GDMT at discharge and ACM were compared among CM categories. The predictors of 1-year ACM were also evaluated. **Results**: From the 388 patients (male: 76%, age: 61 [50–70] years; NT-proBNP: 5286 [2570–9923] pg/mL; ≥2 cardiovascular–kidney–metabolic disease overlap: 46%), a large proportion received GDMT (RASi: 91%; βB: 85%; MRA: 95%; SGLT2i: 59%; triple therapy [TT: RASi+βB+MRA]: 82%; quadruple therapy [QT: TT + SGLT2i]: 54%) at discharge. Multimorbidity was accompanied with a (*p* < 0.05) lower application ratio of RASi (96% vs. 92% vs. 85%; 0–3 vs. 4–6 vs. ≥7 CMs) and βB therapy (94% vs. 85% vs. 78%), while MRA (99% vs. 94% vs. 94%) and SGTL2i use (61% vs. 59% vs. 57%) did not differ (*p* > 0.05). Patients with multimorbidity were less likely to be treated with TT (93% vs. 82% vs. 73%, *p* = 0.001), while no difference was detected in the implementation of QT (56% vs. 54% vs. 50%, *p* = 0.685). The 1-year ACM of patients with an increased burden of CMs was higher (9% vs. 13% vs. 25%, *p* = 0.003). The risk of 1-year ACM was favourably affected by the use of TT/QT and less severe left ventricular systolic dysfunction, while having ≥5 CMs had an unfavourable impact on prognosis. **Conclusions**: According to our real-world analysis, HFrEF patients with an increased burden of CMs can expect a less favourable outcome. However, modern GDMT can even be applied in this patient population, resulting in a significantly improved prognosis. Thus, clinicians should insist on the early, conscious implementation of a prognosis-modifying drug regime in multimorbid HF patients as well.

## 1. Introduction

The prevalence of cardiovascular (CV) and non-cardiovascular (non-CV) comorbidities (CMs) in patients suffering from heart failure (HF) has increased remarkably in recent years; nowadays, at least 50% of HF patients may present with more than five CMs [1,2,3,4]. The importance of non-CV CMs is clearly illustrated by the fact that a significant proportion of HF hospitalisations are triggered by non-CV reasons [5]. Moreover, due to the ageing population, the impact of non-CV CMs is liable to increase in the future [1,6]. Coexisting comorbidities complicate the diagnosis and the management of HF, worsen quality of life, increase the length of hospital stays, increase the financial burden, and result in a worse prognosis [1,2,3,7,8]. Furthermore, the safe application of complex pharmacotherapy in HF with reduced ejection fraction (HFrEF) is also affected by coexisting CMs [9,10,11,12,13].

CMs already played a central role in the 2016 European Society of Cardiology (ESC) HF Guidelines (GLs), which emphasised that their management is a key element of holistic care for HF patients [14]. Correspondingly, the 2021 ESC HF GLs recommend routine screening for CMs, and the latter’s 2023 Focused Update paid special consideration to their management [15,16].

Multimorbid HF patients (defined as having at least two CMs [17]) with increased CM burden are frequently underrepresented in clinical trials; hence, frequently, only the few most common ones are reported, and strict inclusion and exclusion criteria are often used to avoid competing outcome-related risks [18,19]. Accordingly, scientific data on the impact of multimorbidity on the implementation of modern guideline-directed medical therapy (GDMT) and prognosis are lacking and need to be closely investigated.

In our current retrospective, single-centre, observational study, we present an evaluation of the CM burden in a consecutive cohort of real-world patients with HFrEF requiring hospitalisation due to HF, to measure the former’s impact on GDMT application and all-cause mortality, and to investigate the independent predictors of 1-year all-cause mortality.

## 2. Materials and Methods

### 2.1. Study Population and Design

We undertook a retrospective observational study among a consecutive, non-selected group of HFrEF patients requiring hospitalisation for HF between 1 April 2021 and 30 April 2023 in a tertiary referral cardiac centre at the HF Unit of the Department of Adult Cardiology, Gottsegen National Cardiovascular Center. In-hospital mortality formed an exclusion criterion. To avoid redundancy, in the case of multiple hospitalisations during the data collection period, the first event was considered in our analysis.

Sixteen CV- and non-CV CMs were observed (Table S1): coronary artery disease (CAD), hypertension, atrial fibrillation/flutter, severe valvular heart disease (VHD), stroke, and peripheral artery disease (PAD) were considered as CV CMs, while obesity (body mass index [BMI] > 30 kg/m^2^), type I or type II diabetes mellitus (DM), kidney dysfunction (previously diagnosed chronic kidney disease or known, persistently decreased estimated glomerular filtration rate [eGFR] measured in hospital < 60 mL/min/1.73 m^2^), hyperuricaemia, hypo- or hyperthyroidism, sleep-disordered breathing, asthma/chronic obstructive pulmonary disease (COPD), anaemia (haemoglobin measured in hospital <130 g/L in men and <120 g/L in women), iron deficiency (ferritin < 100 μg/L or transferrin saturation [TSAT] < 20%), and dyslipidaemia were the examined non-CV CMs. Based on the number of coexisting CMs, three categories were established: 0–3, 4–6, or ≥7 CMs. The application of neurohormonal antagonist therapy (renin–angiotensin system inhibitor [RASi]: angiotensin-converting enzyme inhibitor [ACEi]/angiotensin receptor neprilysin inhibitor [ARNI] and angiotensin receptor blocker [ARB]; beta-blocker [βB]; mineralocorticoid receptor antagonist [MRA]) and sodium-glucose cotransporter 2 inhibitor (SGLT2i) dapagliflozin/empagliflozin medication at hospital discharge were evaluated according to the CM categories. The presence of cardiovascular–kidney–metabolic (CKM) overlap was also evaluated, considering three CKM categories: atherosclerotic cardiovascular disease (ASCVD: CAD and/or PAD and/or stroke), kidney dysfunction (previously diagnosed chronic kidney disease or known, persistently decreased eGFR measured in hospital <60 mL/min/1.73 m^2^) and type I or type II DM. The impact of CMs on all-cause mortality was also investigated, as well as the independent predictors of 1-year all-cause mortality.

Our study protocol was reviewed and approved by the National Scientific and Ethical Committee of Hungary (approval number: BM/34521-1/2023), and the present study adheres to the ethical principles of the Declaration of Helsinki (1975, revised in 2013). For our observational study, no written informed consent was required as it did not influence the professional medical care of the patients, required no intervention, and utilised only anonymised retrospective data collection.

### 2.2. Statistical Analysis

Clinical data were obtained from our hospital patient management system, while mortality data were acquired through the electronic social insurance number validity documentation interface of the National Health Insurance Fund. Data were documented in an anonymised form in a Microsoft Excel 16.80 spreadsheet (Microsoft Corporation, Redmond, WA, USA), and IBM SPSS Statistics 26.0 (International Business Machines Corporation, Armonk, NY, USA) was used for statistical calculations.

A Shapiro–Wilk normality test was performed to define the distribution of continuous variables. Based on their non-Gaussian distribution, continuous variables are presented as median and interquartile ranges, while categorical variables are shown as absolute numbers and percentages. The characteristics (demographic, haemodynamic, laboratory parameters, medical therapy) of the three CM and CKM categories were compared with the Kruskal–Wallis or Chi-square test (as applicable). The age-related occurrence of the CMs was evaluated with the Kruskal–Wallis test, and the sex-specific prevalence of the CMs was analysed with the Mann–Whitney U test, while GDMT application in these subgroups was examined with the Chi-square test. The medical and device therapy applied at hospital admission and discharge were compared with the McNemar test in the total cohort. All-cause mortality rates were investigated using the Kaplan–Meier method with log-rank test and univariate Cox-regression analysis. The independent predictors of 1-year all-cause mortality were evaluated with uni- and multivariate Cox-regression analysis. Factors of the univariate Cox-regression analysis with a *p*-value < 0.1 and an odds ratio (OR) ≠ 1 were involved in the multivariate model [20]. In the multivariate Cox-regression analysis, factors with a *p*-value < 0.05 and an odds ratio (OR) ≠ 1 were considered independent predictors. Statistical significance was defined as *p* < 0.05.

## 3. Results

### 3.1. Patient Population

Data from a cohort of 388 consecutive, real-world HFrEF patients requiring hospitalisation due to HF were analysed (Table 1). Seventy-six percent of them were male, and the median age was 61 [50–70] years. Forty-one percent of the cohort had been hospitalised for HF previously, and 39% of the total cohort was diagnosed with “de novo” HF.

### 3.2. Multimorbidity in HF

**Figure 1:**
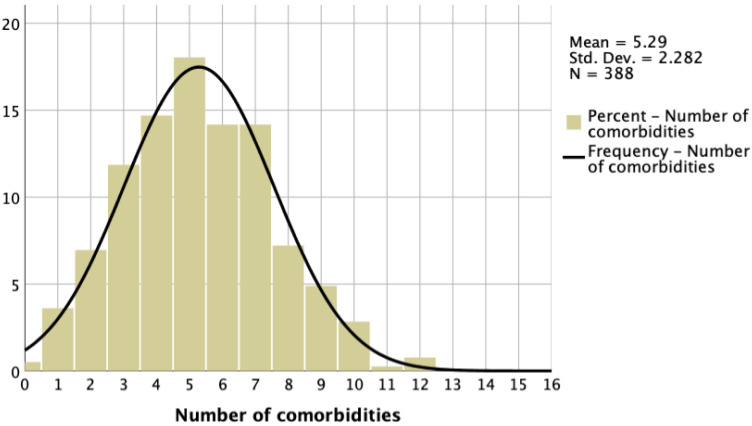
Number of CMs intotal HFrEF patient cohort.

The median number of CMs in the patients was 5 [4–7]; 23% had 0–3, 47% had 4–6, and 30% had ≥7 CMs (Figure 1, Table 2).

The occurrence of the examined CMs is reported in Table 2.

Those with the highest CM burden were characterised by higher age, an increased proportion of female gender, more frequent previous HF hospitalisations, less frequent “de novo” HF diagnosis, higher NT-proBNP levels, and more advanced kidney dysfunction at hospital admission (Table 1). Furthermore, ageing was accompanied by a progressively increased number of (both CV and non-CV) CMs as expected (Figure S1A,B).

Female sex was associated with a growing number of non-CV CMs (Figure S2).

**Figure 2:**
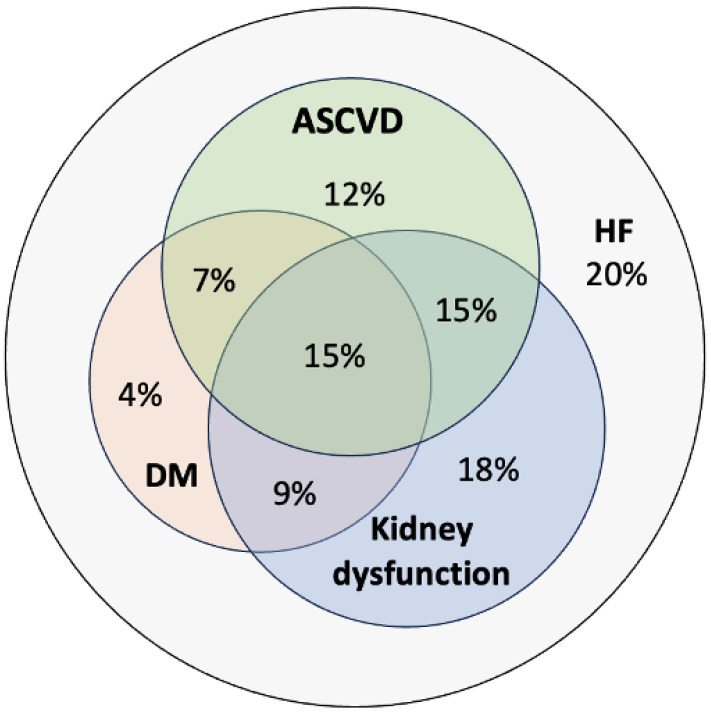
Cardiovascular–kidney–metabolic overlap. ASCVD: atherosclerotic cardiovascular disease; DM: diabetes mellitus; HF: heart failure.

Considering CKM overlap, 20% of the patients had HF without any CKM comorbidities, 34% of the HF patients were affected by 1 CKM disease, while 31% by 2, and 15% by all 3 CKM diseases (Figure 2).

### 3.3. The Effect of Multimorbidity on GDMT Implementation

During hospitalisation, the implementation of neurohormonal antagonist therapy (RASi: 61% vs. 91%; βB: 61% vs. 85%; MRA: 46% vs. 95%; hospital admission vs. hospital discharge) and SGLT2is (34% vs. 59%) increased significantly (*p* < 0.001) thus 82% of the cohort was treated with triple therapy (TT: RASi [ACEi/ARB/ARNI] + βB + MRA), and 54% received quadruple therapy (QT: TT + SGLT2i) at hospital discharge (Table 3).

**Figure 3:**
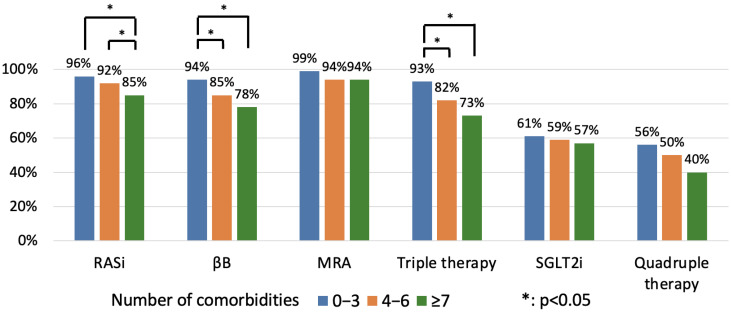
Application of medical therapy at hospital discharge based on number of CMs. MRA: mineralocorticoid receptor antagonist; RASi: renin–angiotensin system inhibitor; SGLT2i: sodium-glucose co-transporter 2 inhibitor; βB: beta-blocker.

At hospital discharge, multimorbidity was accompanied by a significantly lower application ratio of RASi (96% vs. 92% vs. 85%, *p* = 0.017; 0–3 vs. 4–6 vs. ≥7 CMs) and βB therapy (94% vs. 85% vs. 78%, *p* = 0.004), while MRA (99% vs. 94% vs. 94%, *p* = 0.171) and SGTL2i use (61% vs. 59% vs. 57%, *p* = 0.886) did not differ among the CM categories (Figure 3). Patients with sequentially more CMs were less likely to receive TT (93% vs. 82% vs. 73%, *p* = 0.001), while no significant difference was observed in the application ratio of QT (56% vs. 54% vs. 50%, *p* = 0.685).

**Figure 4:**
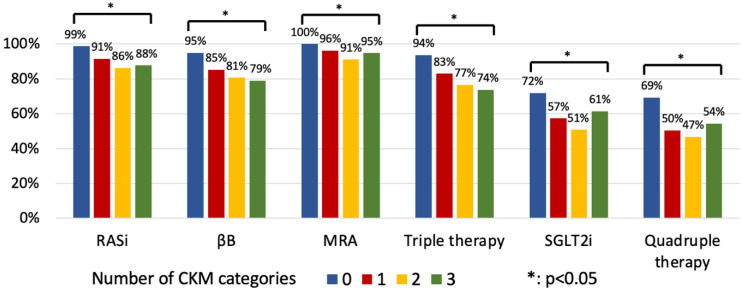
Application of medical therapy at hospital discharge based on CKM overlap. CKM: cardiovascular–kidney–metabolic; MRA: mineralocorticoid receptor antagonist; RASi: renin–angiotensin system inhibitor; SGLT2i: sodium-glucose co-transporter 2 inhibitor; βB: beta-blocker.

The more frequent CKM overlap led to a lower application ratio of GDMT (*p* < 0.05) (Figure 4).

In the age-specific subanalysis, patients aged > 65 years with 4–6 CMs were less likely to receive βB medication (90% vs. 76%, *p* = 0.012; ≤65 years vs. >65 years, respectively), SGLT2is (87% vs. 73%, *p* = 0.016), TT (65% vs. 48%, *p* = 0.033), or QT (61% vs. 42%, *p* = 0.014) compared to the ones aged ≤ 65 years (Table S2). Considering sex categories, female patients with 4–6 CMs received βBs (74% vs. 87%, *p* = 0.047; female sex vs. male sex) less frequently (Table S3).

### 3.4. Effect of Multimorbidity on Prognosis and Predictors of Mortality

**Figure 5:**
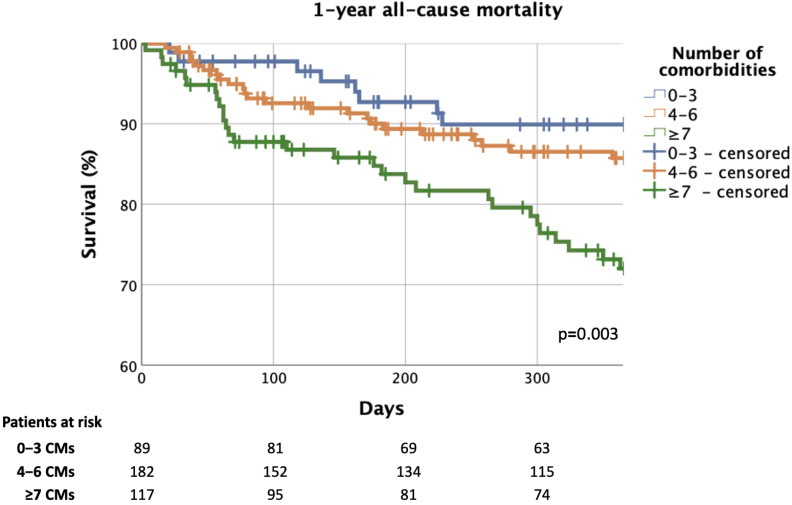
One-year all-cause mortality based on number of CMs. CM: comorbidity.

During the median 437 [179–742] days of follow-up, all-cause mortality was 24% in the total cohort, while 1-year all-cause mortality was 15%. The increasing burden of CMs resulted in notably less favourable survival (13% vs. 19% vs. 41%, *p* < 0.001; 0–3 vs. 4–6 vs. ≥7 CMs) and 1-year all-cause mortality also increased (9% vs. 13% vs. 25%, *p* = 0.003) (Figure 5 and Figure S3). Compared to the ones with the least CM burden, ≥7 CMs gave a threefold risk (hazard ratio [HR] = 3.007, 95% confidence interval [CI] = 1.375–6.579, *p* = 0.006) of 1-year all-cause mortality.

**Figure 6:**
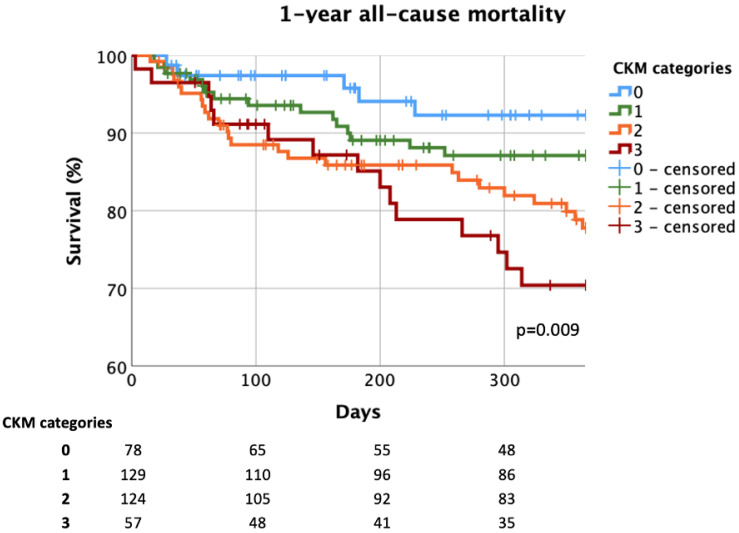
One-year all-cause mortality considering cardiovascular–kidney–metabolic overlap. CKM: cardiovascular–kidney–metabolic.

Advanced CKM disease also led to highly elevated 1-year all-cause mortality rates (6% vs. 12% vs. 20% vs. 26%, *p* = 0.009; 0 vs. 1 vs. 2 vs. 3 CKM) (Figure 6). Patients affected by two CKM diseases, had a threefold risk (HR = 3.056, 95% CI = 1.170–7.983, *p* = 0.023), while the ones with three CKM diseases had a fourfold risk (HR = 4.085, 95% CI = 1.485–11.239, *p* = 0.006) of 1-year all-cause mortality compared to the patients without any CKM overlap.

The results of the univariate Cox-regression analysis are shown in Table 4. In the multivariate model, LVEF (HR = 0.962, 95% CI = 0.927–0.999, *p* = 0.043), the use of TT/QT (HR = 0.391, 95% CI = 0.229–0.669, *p* = 0.001), and the presence of ≥5 CMs (HR = 2.373, 95% CI = 1.133–4.971, *p* = 0.022) were proven to be the independent predictors of 1-year all-cause mortality.

## 4. Discussion

### 4.1. Main Findings

Multimorbidity has severe therapeutic consequences and effects on the prognosis of HFrEF patients. Based on our study among real-world HFrEF patients requiring HF hospitalisation, a growing number of CMs can be expected. According to our analysis, CKM overlap syndrome affects a significant proportion of HFrEF patients. Patients with a greater CM burden are less likely to be treated with GDMT at hospital discharge, even though their all-cause mortality is significantly less favourable. However, according to our results, the implementation and optimisation of GDMT is safe and feasible in most patients in our cohort in everyday practice. Furthermore, based on our multivariate Cox-regression analysis, the application of TT/QT and the less severe left ventricular systolic dysfunction reduces the risk of 1-year all-cause mortality, while the presence of ≥5 CMs is associated with a significant unfavourable effect on survival.

### 4.2. Multimorbidity in HF

With the improvement of complex care and as a consequence of the average increase in life expectancy, a higher CM burden can be expected in HF patients nowadays [1,21,22]. Unquestionably, treating patients with HF goes beyond just focusing on the heart [1,21,23,24,25,26,27,28,29,30]. In 2018, Conrad et al. concluded that the number of CMs in an HF cohort of the United Kingdom had increased from 3.4 (standard deviation [SD] = 1.9) to 5.4 (SD = 2.5) from 2002 to 2014 [4]. In a study from the United States among patients hospitalised for HF in real-world clinical practice, there was an increase in the proportion of patients with coexisting multiple (≥3) non-CV CMs from 18% to 29%, while the share of those presenting without non-CV CMs decreased from 22% to 16% from 2005 to 2014 [31]. Moreover, as Screever et al. reported, over the last 15 years—despite the improvement observed in the available effective treatment options for CMs in HFrEF [32,33,34,35,36,37,38,39]—the negative impact of multimorbidity on HF hospitalisation and all-cause mortality remained significant [40]. We must keep in mind that CMs are frequently not reported in HF clinical trials: according to a systematic review of 118 HF studies, data on each examined CM was documented only in a mean of 35% of the trials [18]. Furthermore, several CMs are frequently underdiagnosed in HF in everyday practice [39,41,42,43].

In our analysis, patients had a median of 5 [4–7] CMs, which exceeded the results of the REPORT-HF trial (60% of patients had ≥2 CMs, while 22% had ≥5 CMs) [17] and the data from the Swedish HF Registry (SwedeHF; the majority of the examined cohort had four CMs; more than 60% of them had at least four CMs) [3]. However, a comparison of these analyses is difficult due to the heterogeneous characteristics of the enrolled patients; it is also worth noting that even though the median age of our patient cohort (61 [50–70] years) was notably younger than that in the SwedeHF Registry (76 [67–82] years), their CM profile was definitely worse [3]. Moreover, the proportion of patients with an increased burden of non-CV CMs in the current analysis (≥3 non-CV CMs: 67%) was higher than reported in the ASCEND-HF trial (≥3 non-CV CMs: 35.7%) [2] and the “high-intensity” care group of the STRONG-HF trial (at least three non-CV CMs: 11.4%) [44].

Our results indicate that iron deficiency, hypertension, and kidney dysfunction were among the most frequent CMs in the examined consecutive, real-world HF cohort. Iron deficiency may affect 50–55% of the chronic HF patient population and be present in as many as 80% of acute HF patients [15,45,46]. Similarly, 74% of our patients were affected by iron deficiency. It has to be emphasised that based on the growing evidence published in recent years, not only screening for iron deficiency but also intravenous iron supplementation in HFrEF and HF with mildly reduced ejection fraction (HFmrEF) is justified according to the 2023 ESC HF GLs [16]. The prevalence of hypertension in our analysis (65%) was comparable with the previously published registries (ESC HF Long-Term Registry Acute HF subgroup: 65.6%; SwedeHF Registry: 64.9%; Hungarian HF Registry: 65.6%) [3,47,48]. The occurrence of kidney dysfunction can reach 30–50% in HF patients [49], and 10% of patients with HFrEF have at least < 30 mL/min/1.73 m^2^ or worse eGFR values [50]. In the ESC HF Long-Term Registry, in the SwedeHF Registry, and an analysis of the PARADIGM-HF and the ATMOSPHERE trials, the most prevalent non-cardiac CMs also involved chronic kidney disease [3,51,52], and in our analysis, 57% of the cohort was affected by at least moderate kidney dysfunction. The occurrence of CKM overlap was similar in our study (20% vs. 34% vs. 31% vs. 15%; 0 vs. 1 vs. 2 vs. 3 CKM, respectively) to the FINEARTS-HF (22.4% vs. 40.6% vs. 28.9% vs. 8.2%), DELIVER (22.2% vs. 38.3% vs. 30.4% vs. 9.1%), PARAGON-HF (22.2% vs. 41.7% vs. 27.9% vs. 8.3%), and TOPCAT (32.3% vs. 39.4% vs. 22.6% vs. 5.8%) trials [53]. The frequent occurrence of dyslipidaemia in our analysis is explained by the CMs associated with the need for lipid-lowering therapy (e.g., CAD: 53.8% vs. 52.4% vs. 37% vs. 42%; ESC HF Long-Term Registry Acute HF subgroup vs. SwedeHF Registry vs. Hungarian HF Registry vs. current analysis, respectively; chronic kidney disease/kidney dysfunction: 25.3% vs. 40.0% vs. 20.2% vs. 57%; DM: 39% vs. 26.1% vs. 33.5% vs. 35%).

### 4.3. The Effect of Multimorbidity on GDMT Implementation

Although several observational analyses were published within recent years focusing on the relationship of multimorbidity and drug therapy application in HFrEF, the effect of the CMs on the novel pharmaceutical options of HFrEF (ARNI, SGLT2i) and the complex modern TT and QT are yet to be investigated [54] (Table 5).

In the recent analysis of the REPORT-HF Registry, although the majority of the examined cohort was treated with the conventional first-line pillars of HFrEF pharmacotherapy, data regarding the use of SGLT2i, TT, and QT were not reported [17]. In the robust retrospective analysis of the SwedeHF Registry, in most of the examined cohort, a RASi (ACE/ARB/ARNi in 85.4%) and a βB (85.4%) were applied; however, the proportion of patients on MRA therapy (35.2%) remained remarkably modest. Moreover, the proportion of patients on SGLT2i, TT, and QT was not reported [3]. According to our study, in the total cohort, the application ratio of GDMT exceeded the results of recently published data from the Get With The Guidelines–Heart Failure (GWTG-HF) Registry as well, as over a similar data collection period, our patients were more frequently treated with TT (35.2% vs. 82%; GTWG-HF Registry vs. current study, respectively), SGLT2i medication (23.5% vs. 59%), and QT (13.0% vs. 54%), while the implementation of ARNI was comparable (27.7 vs. 23%) [55]. Zheng et al. reported an even smaller—14%—application ratio of TT/QT at hospital discharge among hospitalised HFrEF patients [56]. In our analysis, the SGLT2i penetrance of 59% was higher than that of the recently published SwedeHF Registry (37%) [57].

In our analysis, the mean daily dose of MRA at hospital discharge (treatment group: mean daily dose of spironolactone/eplerenone: 44 mg), approached or even exceeded the relating data of RALES (treatment group: mean daily dose of spironolactone: 26 mg) [58], EPHESUS (treatment group: mean daily dose of eplerenone: 42.6 mg) [59], and EMPHASIS-HF (treatment group: mean daily dose of eplerenone: 39.1 ± 13.8 mg) [60] RCTs. This phenomenon might be explained by the positive effect of novel HFrEF pharmacotherapies as SGLT2i and ARNI medications on reducing the risk of severe hyperkalaemia events [61,62,63].

According to the data from the ESC HF Long-Term Registry, the application of the conventional strategic agents of HFrEF improved during hospitalisation, while the increasing burden of CMs negatively modified GDMT implementation (at discharge, ACEi/ARB in 84.3%, ARNI in 1.7%, βB in 83%, and MRA in 62.3% were applied among patients with 0 non-CV CMs; while among patients with ≥4 non-CV CMs ACEi/ARB in 65.2%, ARNI in 1.1%, βB in 63.4%, MRA in 41.2% was implemented) [51]. However, the proportion of patients on these foundational pillars of HFrEF in this analysis remained remarkably low compared to those reported in recent RCTs [51,63,64]. Furthermore, the proportion of patients on SGLT2is was not documented in this analysis [51].

**Table 5:** Occurrence of CMs and application ratio of GDMT in large HF registries in recent years.

	ESC HF Long-Term Registry (2023) [51]	REPORT-HF (2023) [17]	GTWG-HF (2018) [31]	ASIAN-HF (2019) [65]	ASCEND-HF (2020) [2]	STRONG-HF (2022) [44]	SwedeHF (2023) [3]	Current Analysis (2024)
Number of CMs	not documented	0: 5.4% 1: 13.0% 2: 18.6% 3: 21.2% 4: 17.8% ≥5: 24.0%	not documented	median [IQR]: 3 [2–4]	not documented	not reported	0: 1.9% 1: 7.2% 2: 13.0% 3: 16.8% 4:18.1% 5: 16.4% 6: 12.5 ≥7: 14.1%	median [IQR]: 5 [4–7] 0: 1% 1: 4% 2: 7% 3: 12% 4: 15% 5: 18% 6: 14% 7:14% ≥8: 15%
Number of non-CV/non-cardiac CMs	0: 20.5% 1: 28.7% 2: 23% 3: 15.4% ≥4: 12.5%	not reported	0: 18% 1: 30% 2: 27% ≥3: 25%	not reported	mean ± SD: 2.2 ± 1.37 0: 8.9% 1: 25.3% 2: 30.0% 3: 20.1% ≥4: 15.6%	0: 24.3% 1: 39.8% 2: 24.5% ≥3: 11.4%	0: 14.8% 1: 26.4% 2: 26.1% 3: 18.3% 4: 23% ≥5: 14.4%	median [IQR]: 3 [2–4] 0: 2% 1: 10% 2: 21% 3: 21% 4: 23% ≥5: 23%
RASi	total cohort: not documented 0 non-CV CMs: 86% ≥4 non-CV CMs: 66.3%	total cohort: not reported 0 CMs: 78% * ≥4 CMs: 62% * * not exact values, estimated based on published diagram	not documented	73.7%	total cohort: 60.6%	“High-intensity care” group at day 180: 97.2%	85.4%	91%
βB	total cohort: not documented 0 non-CV CMs: 83.1% ≥4 non-CV CMs: 63.4%	total cohort: not reported 0 CMs: 72% * ≥4 CMs: 56% * * not exact values, estimated based on published diagram	not documented	75.7%	total cohort: 58.1%	“High-intensity care” group at day 180: 95.7%	88.4%	85%
MRA	total cohort: not documented 0 non-CV CMs: 62.3% ≥4 non-CV CMs: 41.2%	total cohort: not reported 0 CMs: 80% * ≥4 CMs: 80% * * not exact values, estimated based on published diagram	not documented	52.1%	total cohort: 27.8%	“High-intensity care” group at day 180: 95.7%	35.2%	95%
TT	not documented	not reported	not documented	not reported	not documented	not reported	not documented	82%
SGLT2i	not documented	not reported	not documented	not reported	not documented	not reported	not documented	59%
QT	not documented	not reported	not documented	not reported	not documented	not reported	not documented	54%

CM: comorbidity; GDMT: guideline-directed medical therapy; ESC: European Society of Cardiology; HF: heart failure; IQR: interquartile range; GWTG-HF: Get With The Guidelines–Heart Failure Registry; MRA: mineralocorticoid receptor antagonist; non-CV: non-cardiovascular; QT: quadruple therapy; RASi: renin–angiotensin system inhibitor; SD: standard deviation; SGLT2i: sodium-glucose co-transporter 2 inhibitor; SwedeHF: Swedish Heart Failure Registry; TT: triple therapy; βB: beta-blocker.

To best of our knowledge, the current analysis is among the first to provide data on the impact of CMs on the modern TT/QT of HFrEF. Our results confirmed that patients with a greater CM burden were less likely to be treated with GDMT at hospital discharge (TT: 93% vs. 82% vs. 73%, *p* = 0.001; 0–3 vs. 4–6 vs. ≥7 CMs), and underscored the strategic importance of the highly prevalent CKM overlap [66], which unfavourably modified the implementation ratio of GDMT (TT: 94% vs. 83% vs. 77% vs. 74%; QT: 69% vs. 50% vs. 47% vs. 54%, *p* < 0.05; 0 vs. 1 vs. 2 vs. 3 CKM). However, the proportion of patients on TT and QT remained remarkably high even among those with an increased number of CMs (patients with ≥7 CMs: RASi: 85%; βB: 78%; MRA: 94%; TT: 73%; SGLT2i: 57%; QT: 50%) and CKM syndrome (3 CKM overlap syndromes: RASi: 88%; βB: 79%; MRA: 95%; TT: 74%; SGLT2i: 61%; QT: 54%). Our results demonstrated the widespread applicability of SGLT2i therapy regardless of CM burden (SGLT2i: 72% vs. 57% vs. 51% vs. 61%, *p* < 0.05; 0 vs. 1 vs. 2 vs. 3 CKM). Besides HF, the beneficial effect of SGLT2i dapa- and empagliflozin in diabetes and chronic kidney disease are well established (DECLARE-TIMI 58 [35], EMPA-REG OUTCOME [34], DAPA-CKD [67], EMPA-KIDNEY [33]). In the recently published analysis of the EMPA-KIDNEY trial, empagliflozin reduced the risk of the composite endpoint of kidney disease progression or CV death by 28% (HR = 0.72, 95% CI = 0.64–0.82) regardless of the presence of multimorbidity (defined at least eight CMs) [68]. Moreover, in the post hoc analysis of the IRONMAN trial, there was a favourable trend to a greater increase in haemoglobin level with ferric derisomaltose in HF patients receiving SGLT2i medications [69]. The potential beneficial effect of SGLT2is on iron homeostasis may be related to the improved hepcidin production, although, the exact role of SGLT2is in the pathophysiology is not fully understood [70]. The shared analysis of the PARADIGM-HF, DAPA-HF, EMPREROR-Reduced, VICTORIA, and GALACTIC-HF trials also confirmed that even in more severe patient subgroups with frequent CMs as chronic kidney disease or diabetes unquestionably benefit from GDMT of HF [71].

The negative impact of CMs on GDMT implementation was noted in the SwedeHF Registry as well [3]. As Tomasoni et al. concluded, those HF patients with >6 CMs were treated with conventional TT in significantly smaller proportions [3]. In the REPORT-HF study, HFrEF patients with a greater CM burden were also less likely to receive prognosis-modifying ACEi/ARB and MRA medications [17]. However, it is well-known from the literature that the prognosis-modifying effect of the first-line pharmacotherapy of HFrEF can be detected within a few weeks after initiation [63,72,73,74,75,76,77] and a growing number of evidence was published recently regarding the effective treatment options of CKM overlap syndrome [66,78,79]; hence, as the current analysis has highlighted, clinicians should insist on the early implementation of GDMT among HFrEF patients with multimorbidity as well.

### 4.4. The Effect of Multimorbidity on Prognosis and the Predictors of Mortality

Multimorbidity has a striking effect on morbidity and mortality in HF patients, even on short-term prognosis [2,78]. In the REPORT-HF, patients with ≥5 CMs had a 1-year all-cause mortality of 26% [17], while the 1-year all-cause mortality of patients with >10 CMs reached 47% in the SwedeHF Registry [3]. In the current study, patients with the greatest CM burden had a 1-year all-cause mortality of 25%, almost three times higher than those with 0–3 CMs. In the ESC HF Long-Term Registry, ≥5 CMs (adjusted HR = 4.0, 95% CI = 3.0–5.3, *p* < 0.001) [51], in the SwedeHF Registry, >6 CMs (adjusted HR = 4.41, 95% CI = 3.77–5.16, *p* < 0.001) [3] increased the risk of all-cause mortality of HFrEF patients nearly fourfold compared to those with no CMs. In the ASCEND-HF trial, even 180-day all-cause mortality was significantly affected independently by ≥4 CMs (adjusted odds ratio [OR] = 2.13, 95% CI = 1.33–3.43, *p* = 0.0017) [2]. Even though in the “high-intensity care” group of the STRONG-HF trial, the proportion of patients treated with target doses of GDMT did not differ significantly regardless of the number of non-cardiac CMs, the risk of the occurrence of the primary endpoint (180-day all-cause mortality/HF readmission) was more than twice as high in those with ≥3 non-cardiac CMs (26.2%) than those without any (10.0%) [44]. This should raise awareness of the importance of the early application of prognosis-modifying therapeutic approaches, even in these patients at high risk of worse outcomes.

Based on the multivariate Cox-regression analysis of our patient cohort, the presence of ≥5 CMs (HR = 2.373, 95% CI = 1.113–4.971, *p* = 0.022) and decreased LVEF (HR = 0.962, 95% CI = 0.927–0.999, *p* = 0.043) led to a less favourable 1-year mortality, while the risk of 1-year all-cause mortality was reduced by the application of TT/QT in proportions of 61% (HR = 0.391, 95% CI = 0.229–0.669, *p* = 0.001).

The 1-year mortality of patients not receiving TT/QT may be more than twice that of patients receiving TT/QT [80]. A meta-analysis by Tromp et al. highlighted that while conventional triple therapy may reduce overall mortality in HFrEF patients by 48%, modern QT can result in a 61% reduction in risk compared to the placebo arm [81]. Vaduganathan et al. revealed in their cross-trial analysis that the application of QT can lead to a survival benefit of several years in excess of that associated with conventional treatment [82].

Similarly to our analysis, in the ESC HF Long-Term Registry, the severity of left ventricular systolic dysfunction was identified as an independent predictor of all-cause mortality among patients hospitalised for acute HF (per 5% increase in LVEF: HR = 0.94, 95% CI = 0.91–0.97, *p* < 0.0001) [47]. The CHARM Programme also emphasised the prognostic importance of LVEF on all-cause mortality in chronic HF patients (every 5% decrease in LVEF below 45%: HR = 1.14, 95% CI = 1.12–1.17) [83].

## 5. Conclusions

HFrEF patients with a greater CM burden may have a less favourable prognosis, which underlines the importance of the implementation of prognosis-modifying GDMT. Our results confirm that although GDMT application among multimorbid HFrEF patients is notably lower, the proportion of patients on TT/QT remains high, even among those with a high CM and CKM burden. According to the multivariate analysis, multimorbidity and more severe left ventricular systolic dysfunction are proven to negatively influence prognosis, while applying TT/QT is an independent positive predictor of survival. Our study should call clinicians’ attention to the relevance of the implementation of GDMT in multimorbid HF patients in daily clinical practice.

### Limitations

The real-world patient population of our single-centre study consisted exclusively of individuals of the Caucasian race, so our results and conclusions cannot be applied with certainty to those outside this group. The analysis included only patients from our institute and solely from a cardiology department, which increases potential selection bias. Considering the retrospective nature of our analysis, only known CMs could be assessed. As for the de novo diagnosis of CMs at admission, the available data were applied. For this reason, it was not possible to use a validated CM score, such as the Charlson or Elixhauser CM Index. CMs were only included at the time of cohort entry and not examined during the follow-up period, which may influence outcomes. Hence, the severity of CMs at baseline and disease progression over time (e.g., glycaemic control in DM patients) may also significantly impact outcome (rather than the exact number of CMs alone). The measurement of urine albumin creatinine ratio was not used widely in daily clinical practice at the time of the current analysis. Although a broad range of CMs were examined in our study, the role of unmeasured, undiagnosed CMs on GDMT application and prognosis cannot be ruled out. Regarding the effect of CMs on GDMT application and prognosis, it has to be highlighted that there is no universally accepted CM-based categorisation approach, as the number and type of CMs vary in the literature. A further limitation is the size of the study cohort. The age- and sex-related subanalyses are of limited value due to the small number of cases within each category. The use of SGLT2is and ARNI was affected by the reimbursement rules in Hungary during the time of the analysis.

## Figures and Tables

**Table 1:** Main characteristics of cohort.

Parameters	Total Cohort n = 388	0–3 CMs n = 89	4–6 CMs n = 182	≥7 CMs n = 117	*p*-Value
Male gender (%)	76	73	81	68	0.033
Age, median [IQR], years	61 [50–70]	52 [44–63]	60 [50–71]	68 [60–75]	<0.001
Previous hospitalisation primarily due to HF (%)	41	29	36	60	<0.001
De novo HF (%)	39	51	41	19	<0.001
LVEF, median [IQR], %	25 [20–30]	24 [19–29]	25 [20–30]	26 [20–31]	0.474
Heart rate, median [IQR], min^−1^	90 [76–108]	92 [76–100]	95 [79–112]	84 [73–102]	0.017
Systolic blood pressure, median [IQR], mmHg	120 [110–139]	120 [110–133]	124 [110–143]	116 [104–137]	0.092
Laboratory parameters at admission					
creatinine, median [IQR], μmol/L	113 [89–136]	91 [77–104]	108 [86–130]	134 [115–177]	<0.001
eGFR, median [IQR], mL/min/1.73 m^2^	57 [46–74]	72 [60–86]	59 [51–75]	44 [33–54]	<0.001
potassium, median [IQR], mmol/L	4.1 [3.9–4.5]	4.1 [3.9–4.4]	4.2 [3.9–4.5]	4.1 [3.9–4.6]	0.482
sodium, median [IQR], mmol/L	138 [135–140]	138 [137–140]	137 [135–140]	138 [135–140]	0.135
haemoglobin, median [IQR], g/L	140 [124–152]	146 [137–158]	140 [127–154]	130 [114–143]	<0.001
NT-proBNP, median [IQR], pg/mL	5286 [2570–9923]	4209 [1912–9058]	4986 [2507–9365]	6477 [3600–14,736]	0.004

CM: comorbidity; eGFR: estimated glomerular filtration rate; HF: heart failure; IQR: interquartile range; LVEF: left ventricular ejection fraction; NT-proBNP: N-terminal pro-B type natriuretic peptide.

**Table 2:** CM burden of investigated cohort.

Parameters	Total Cohort n = 388
CV CMs	
Hypertension (%)	65
Atrial fibrillation/flutter (%)	44
CAD (%)	42
VHD (%)	20
Stroke (%)	10
PAD (%)	8
Non-CV CMs	
Dyslipidaemia (%)	75
Iron deficiency (%)	74
Kidney dysfunction (%)	57
Obesity (%)	35
DM (%)	35
Hyperuricaemia (%)	34
Anaemia (%)	22
Hypo-/hyperthyroidism (%)	17
Asthma/COPD (%)	17
Sleep-disordered breathing (%)	2
Number of CMs, median [IQR]	5 [4–7]
≥1 CMs (%)	99
≥2 CMs (%)	96
≥3 CMs (%)	89
≥4 CMs (%)	77
≥5 CMs (%)	62
≥6 CMs (%)	44
≥7 CMs (%)	30
≥8 CMs (%)	16
≥9 CMs (%)	9
Number of CV CMs, median [IQR]	2 [1–3]
≥1 CV CMs (%)	90
≥2 CV CMs (%)	60
≥3 CV CMs (%)	28
≥4 CV CMs (%)	9
Number of non-CV CMs, median [IQR]	3 [2–4]
≥1 non-CV CMs (%)	98
≥2 non-CV CMs (%)	88
≥3 non-CV CMs (%)	67
≥4 non-CV CMs (%)	46
≥5 non-CV CMs (%)	24
≥6 non-CV CMs (%)	12

CAD: coronary artery disease; CM: comorbidity; COPD: chronic obstructive pulmonary disease; CV: cardiovascular; DM: diabetes mellitus; IQR: interquartile range; non-CV: non-cardiovascular; PAD: peripheral artery disease; VHD: valvular heart disease.

**Table 3:** Medical and device therapy at hospital admission and discharge.

Medical and Device Therapy	At Admission n = 388	At Discharge n = 388	*p*-Value
RASi (%)	61	91	<0.001
ACEi/ARB (%)	46	68	<0.001
ARNI (%)	15	23	<0.001
βB (%)	61	85	<0.001
MRA (%)	46	95	<0.001
TT (%)	14	82	<0.001
SGLT2i (%)	34	59	<0.001
QT (%)	9	54	<0.001
TD RASi (%)	16	18	0.644
TD ACEi/ARB (%)	11	15	0.229
TD ARNI (%)	5	3	0.031
TD βB (%)	14	7	<0.001
TD MRA (%)	18	65	<0.001
TD TT (%)	3	2	0.727
TD QT (%)	2	2	1.000
CRT-P/CRT-D (%)	12	16	<0.001
ICD (except for CRT-D) (%)	10	15	<0.001

ACEi: angiotensin-converting enzyme inhibitor; ARB: angiotensin receptor blocker; ARNI: angiotensin receptor neprilysin inhibitor; CRT-D/CRT-P: cardiac resynchronisation therapy with or without defibrillator; ICD: implantable cardioverter defibrillator; MRA: mineralocorticoid receptor antagonist; QT: quadruple therapy; RASi: renin–angiotensin system inhibitor; SGLT2i: sodium-glucose co-transporter 2 inhibitor; TD: target dose; TT: triple therapy; βB: beta-blocker.

**Table 4:** Predictors of 1-year all-cause mortality.

**1-Year All-Cause Mortality—Univariate Cox-Regression**				
	**HR**	**95% CI**		***p*-Value**
Age (/1 year)	1.031	1.011	1.052	0.003
Female gender	0.816	0.434	1.537	0.530
Heart rate (/1 min^−1^)	0.992	0.980	1.003	0.158
Systolic blood pressure (/1 mmHg)	0.987	0.975	0.998	0.024
Potassium at discharge > 4.5 mmol/L	1.249	0.713	2.191	0.437
LVEF (/1%)	0.958	0.925	0.993	0.018
De novo HF	0.341	0.177	0.657	0.001
TT/QT at discharge	0.290	0.173	0.489	<0.001
CRT at discharge	2.119	1.208	3.715	0.009
≥5 CMs	3.005	1.562	5.780	0.001
CAD	1.079	0.650	1.793	0.768
Hypertension	0.939	0.555	1.587	0.813
Atrial fibrillation/flutter	2.088	1.249	3.490	0.005
VHD	2.804	1.666	4.721	<0.001
Stroke	1.736	0.854	3.527	0.127
PAD	1.976	0.939	4.161	0.073
Obesity	0.468	0.241	0.908	0.025
DM	2.077	1.251	3.446	0.005
Kidney dysfunction	2.217	1.236	3.977	0.008
Hyperuricaemia	0.939	0.555	1.587	0.813
Hypo-/hyperthyroidism	2.088	1.249	3.490	0.005
Sleep-disordered breathing	2.217	1.236	3.977	0.008
Asthma/COPD	1.201	0.713	2.021	0.491
Anaemia	1.246	0.662	2.346	0.496
Iron deficiency	1.776	0.787	4.005	0.167
Dyslipidaemia	1.209	0.665	2.200	0.534
**1-Year All-Cause Mortality—Multivariate Cox-Regression**				
	**Adjusted HR**	**95% CI**		***p*-Value**
Age (/1 year)	1.021	0.999	1.044	0.066
Systolic blood pressure (/1 mmHg)	0.991	0.980	1.002	0.116
**LVEF (/1%)**	**0.962**	**0.927**	**0.999**	**0.043**
De novo HF	0.499	0.241	1.031	0.060
**TT/QT at discharge**	**0.391**	**0.229**	**0.669**	**0.001**
CRT at discharge	1.217	0.664	2.229	0.525
**≥5 CMs**	**2.373**	**1.133**	**4.971**	**0.022**

CAD: coronary artery disease; CI: confidence interval; CM: comorbidity; COPD: chronic obstructive pulmonary disease; CRT: cardiac resynchronisation therapy; DM: diabetes mellitus; HF: heart failure; HR: hazard ratio; LVEF: left ventricular ejection fraction; QT: quadruple therapy; PAD: peripheral artery disease; TT: triple therapy; VHD: valvular heart disease.

## Data Availability

All data generated or analysed during this study are included in this article. Anonymised data of the study cohort can be claimed at a reasonable request. The formal request must specify the exact purpose for which the data will be used, considering the ethical standards. The enquiries can be directed to the corresponding author (Balázs Muk; e-mail address: balazsmukmd@gmail.com), who forwards the request to an independent review committee. Access is provided by the independent review committee. The data-sharing agreement must be signed. The anonymised data of the cohort will be provided in a secure data-sharing environment. Researchers whose proposed use of the data was approved can access the data, and the data can be used for the approved specific purposes.

## Supplementary Materials

The following supporting information can be downloaded at: https://www.mdpi.com/article/10.3390/jcm14010139/s1. Table S1: Examined CV and non-CV CMs; Table S2: GDMT application according to age categories; Table S3: GDMT application according to sex categories; Figure S1: (A) Median number of CMs according to age categories; (B) Median number of CMs comparing patients aged ≤ 65 years and >65 years; Figure S2: Median number of CMs comparing sex categories; Figure S3: Correlation between prevalence of CMs and 1-year all-cause mortality.
