# Supplementary material for: Therapeutic Consequences and Prognostic Impact of Multimorbidity in Heart Failure: Time to Act
Source: J Clin Med. 2024 Dec 29;14(1):139. doi: 10.3390/jcm14010139 (PMC11722306; doi:10.3390/jcm14010139)
Supplement: Supplementary file 1 [file jcm-14-00139-s001.zip › jcm-3368008-supplementary.pdf]

Supplementary material

Therapeutic Consequences and Prognostic Impact of Multimorbidity in Heart Failure: Time to Act

Fanni Bánfi-Bacsárdi, Ádám Kazay, Tamás G. Gergely, Zsolt Forrai, Tamás Péter Füzesi, Laura Fanni Hanuska, Pál Péter Schäffer, Dávid Pilecky, Máté Vámos, Vivien Vértes, Miklós Dékány, Péter Andréka, Zsolt Piróth, Noémi Nyolczas, Balázs Muk

Table S1. Examined CV and non-CV CMs

CV CMs	Non-CV CMs
CAD	Obesity
Hypertension	DM
Atrial fibrillation/flutter	Kidney dysfunction
Severe VHD	Hyperuricaemia
Stroke	Hypo-/hyperthyroidism
PAD	Sleep-disordered breathing
	Asthma/COPD
	Anaemia
	Iron deficiency
	Dyslipidaemia

CAD: coronary artery disease; CM: comorbidity; COPD: chronic obstructive pulmonary disease; CV: cardiovascular; DM: diabetes mellitus; non-CV: non-cardiovascular; PAD: peripheral artery disease; VHD: valvular heart disease.

Table S2. GDMT application according to age categories

Parameters	0-3 CMs			4-6 CMs			≥7 CMs		
	**≤ 65 years n = 72**	**> 65 years n = 17**	***p-value***	**≤ 65 years n = 116**	**> 65 years n = 66**	***p-value***	**≤ 65 years n = 51**	**> 65 years n = 66**	***p-value***
RASi (%)	94	100	0.320	93	91	0.593	88	82	0.340
βB (%)	93	100	0.263	90	76	0.012	78	77	0.881
MRA (%)	99	100	0.625	96	91	0.193	98	91	0.107
TT (%)	58	71	0.352	65	48	0.033	59	56	0.765
SGLT2i (%)	92	100	0.218	87	73	0.016	75	71	0.692
QT (%)	93	71	0.183	61	42	0.014	49	52	0.789

CM: comorbidity; MRA: mineralocorticoid receptor antagonist; QT: quadruple therapy; RASi: renin-angiotensin system inhibitor; SGLT2i: sodium-glucose co-transporter 2 inhibitor; TT: triple therapy; βB: beta-blocker.

Table S3. GDMT application according to sex categories

Parameters	0-3 CMs			4-6 CMs			≥7 CMs		
	**Female n=24**	**Male n=65**	*p-value*	**Female n=34**	**Male n=148**	*p-value*	**Female n=37**	**Male n=80**	*p-value*
RASi (%)	96	95	0.928	91	93	0.784	81	86	0.471
βB (%)	88	97	0.087	74	87	0.047	84	75	0.288
MRA (%)	100	98	0.541	97	93	0.400	95	94	0.858
TT (%)	75	55	0.093	56	59	0.702	59	56	0.744
SGLT2i (%)	88	95	0.188	74	84	0.162	73	73	0.957
QT (%)	67	52	0.226	47	56	0.341	54	49	0.594

CM: comorbidity; MRA: mineralocorticoid receptor antagonist; QT: quadruple therapy; RASi: renin-angiotensin system inhibitor; SGLT2i: sodium-glucose co-transporter 2 inhibitor; TT: triple therapy; βB: beta-blocker.

Figure S1 A. Median number of CMs according to age categories

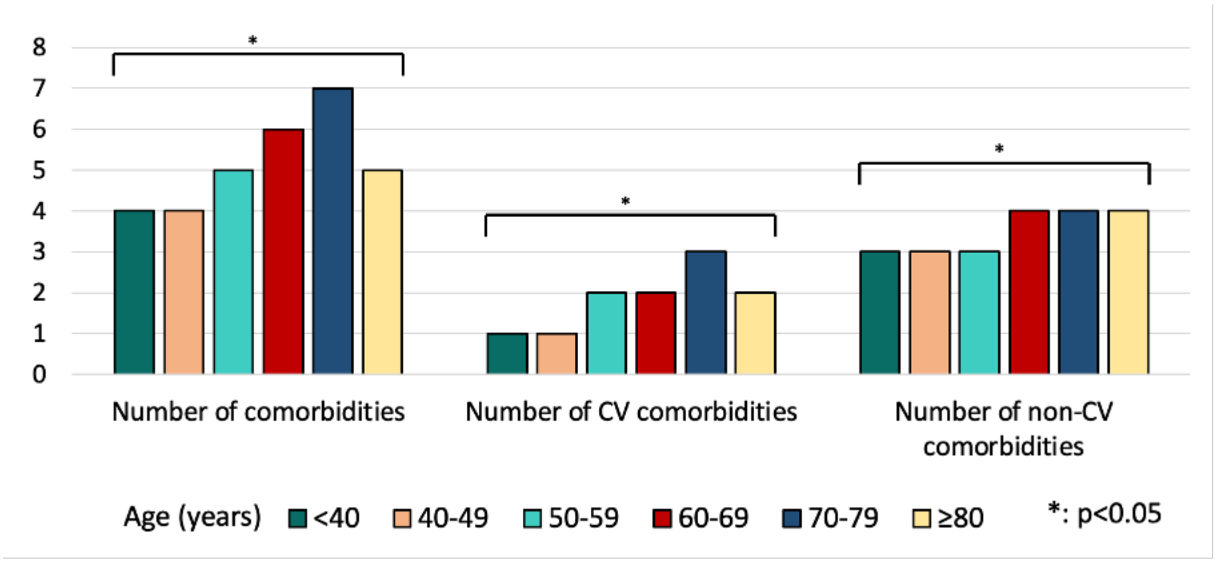

CM: comorbidity; CV: cardiovascular; non-CV: non-cardiovascular.

B. Median number of CMs comparing patients aged ≤ 65 years and >65 years

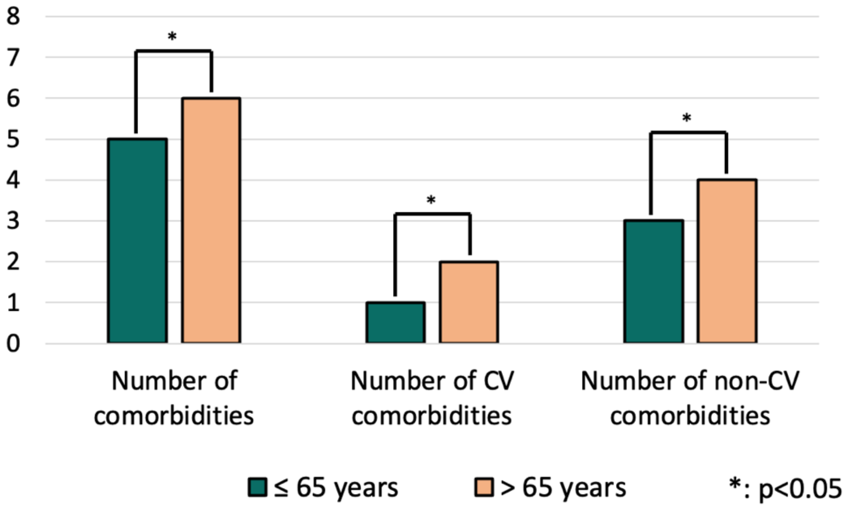

CM: comorbidity; CV: cardiovascular; non-CV: non-cardiovascular.

Figure S2. Median number of CMs comparing sex categories

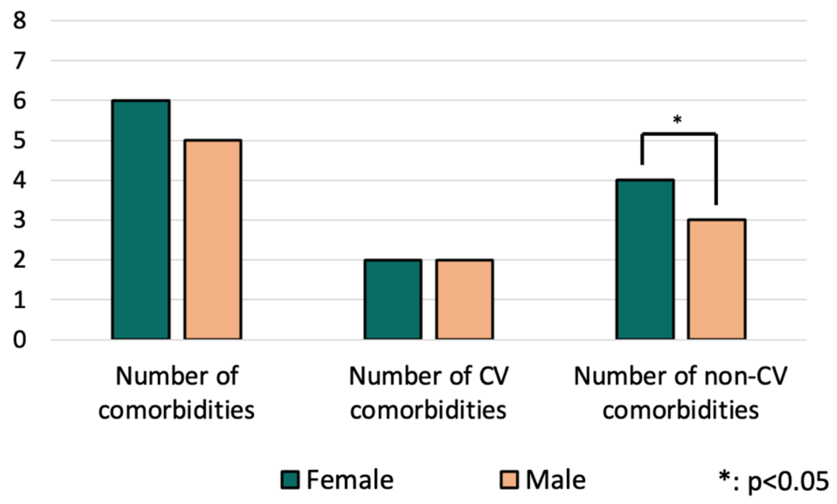

CM: comorbidity; CV: cardiovascular; non-CV: non-cardiovascular.

Figure S3. Correlation between prevalence of CMs and 1-year all-cause mortality

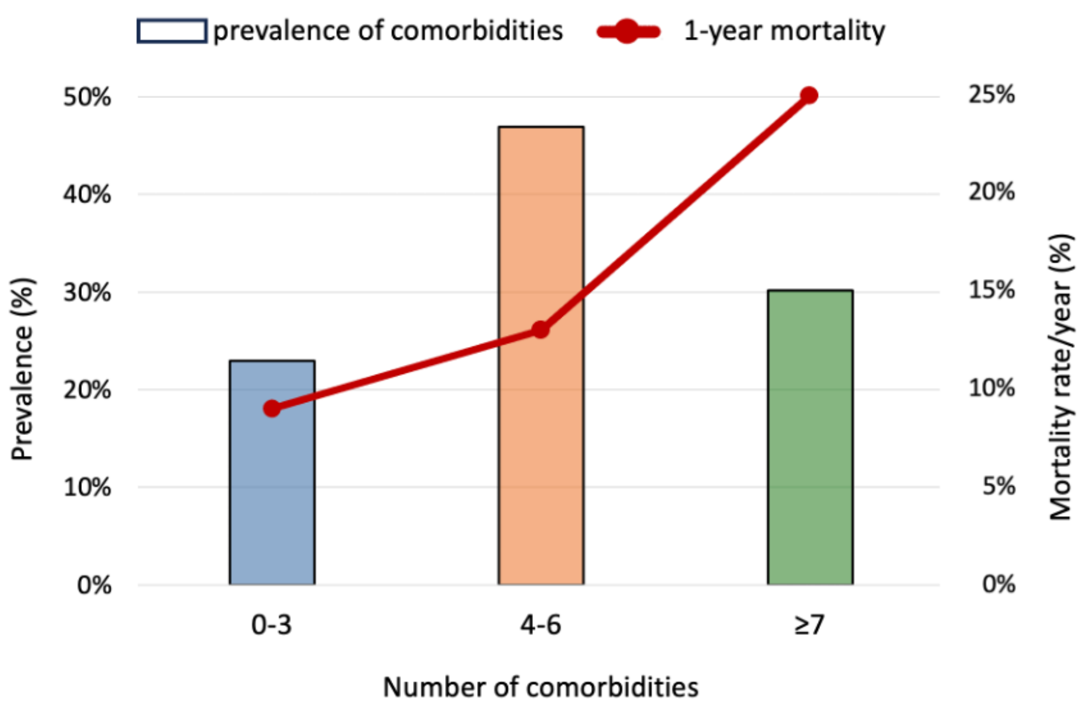

CM: comorbidity.
